# Fine Tuning of Spatial Arrangement of Enzymes in a PCNA-Mediated Multienzyme Complex Using a Rigid Poly-L-Proline Linker

**DOI:** 10.1371/journal.pone.0075114

**Published:** 2013-09-05

**Authors:** Tomoaki Haga, Hidehiko Hirakawa, Teruyuki Nagamune

**Affiliations:** 1 Department of Chemistry and Biotechnology, School of Engineering, the University of Tokyo, Hongo, Tokyo, Japan; 2 Department of Bioengineering, School of Engineering, the University of Tokyo, Hongo, Tokyo, Japan; Center for Genomic Regulation, Spain

## Abstract

Inspired by natural multienzyme complexes, many types of artificial multienzyme complexes have recently been constructed. We previously constructed a self-assembled complex of a bacterial cytochrome P450 and its ferredoxin and ferredoxin reductase partners using heterotrimerization of proliferating cell nuclear antigen (PCNA) from *Sulfolobus solfataricus*. In this study, we inserted different peptide linkers between ferredoxin and the PCNA subunit, and examined the effect on activity of the self-assembled multienzyme complex. Although the activity was affected by the lengths of both the rigid poly-L-proline-rich linkers and the flexible Gly_4_-Ser repeating linkers, the poly-L-proline-rich linkers provided the greatest activity enhancement. The optimized poly-L-proline-rich linker enhanced the activity 1.9-fold compared with the GGGGSLVPRGSGGGGS linker used in the previously reported complex, while the Gly_4_-Ser repeating linkers, (G_4_S)_n_ (n = 1–6), did not yield higher activity than the maximum activity by the optimized poly-L-proline linker. Both the rigidity/flexibility and length of the peptide linker were found to be important for enhancing the overall activity of the multienzyme complex.

## Introduction

In nature, enzymes often work together in cascade or coupled reactions by forming multienzyme complexes. This helps to prevent degradation of unstable intermediate products and to enhance mass transfer between enzymes. Inspired by natural multienzyme complexes, artificial multienzyme complexes have been constructed by gene fusion, co-immobilization, co-entrapment, and scaffold-mediated assembly [1]. Scaffold-mediated multienzyme complexes have been constructed using biomaterials such as RNA [2], DNA [3–7] and protein [8–12]. In these complexes, enzymes are connected to single scaffolds by nucleic acids or peptide linkers. The linkers regulate the relative spatial arrangements of the enzymes, which affect the overall activities of the multienzyme complexes. However, except for a few reports [8,9], the linkers in artificial multienzyme complexes have not been examined in detail.

**Figure 1:**
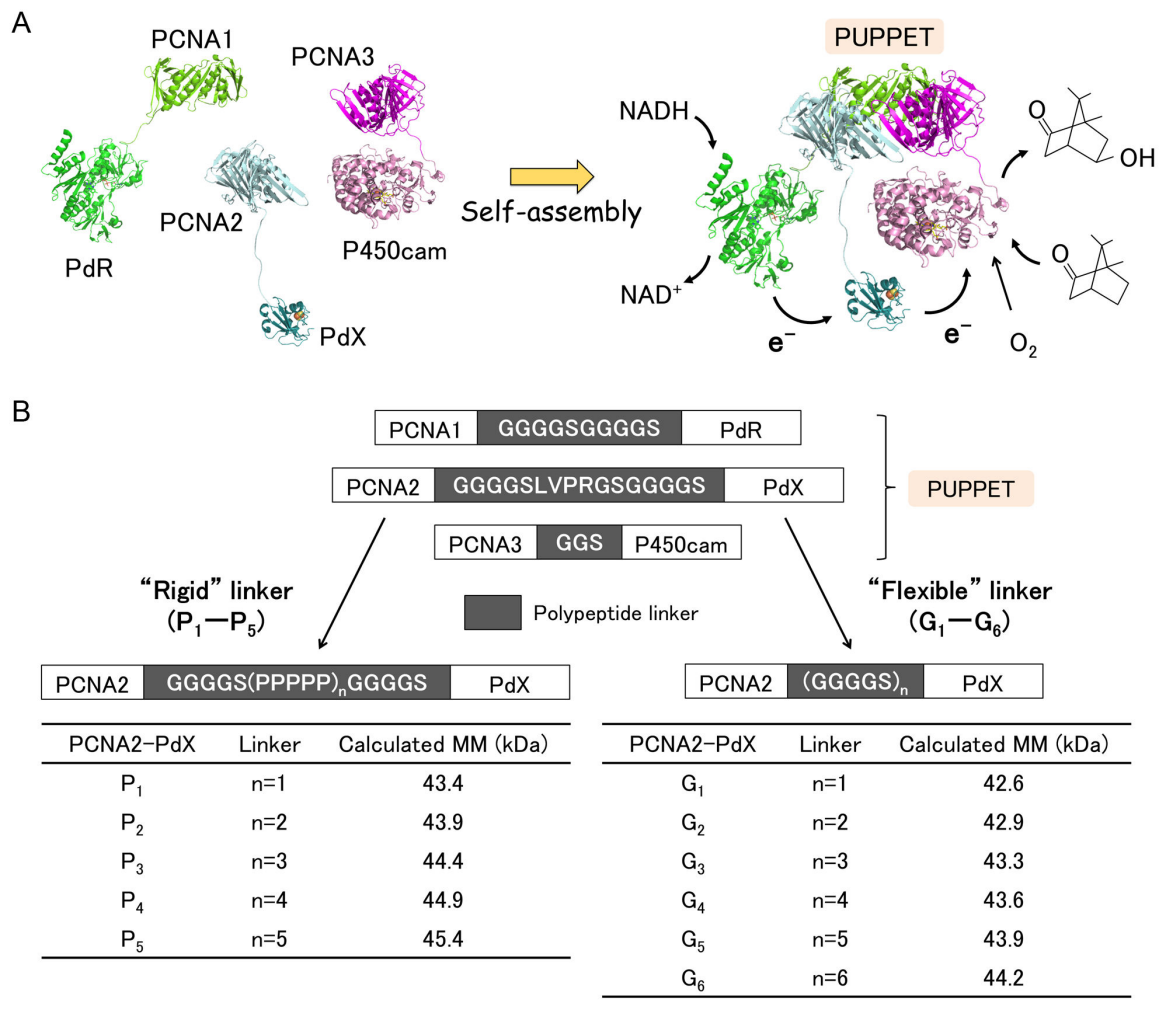
Optimization of the PCNA2-PdX fusion protein linker in PUPPET. (A) Model depicting the self-assembly of PCNA1-PdR, PCNA2-PdX and PCNA3-P450cam. (B) Schematic representation of 11 PCNA2-PdX fusion proteins (P_1_-P_5_ and G_1_-G_6_). The calculated molecular masses of the PCNA2-PdX linker variants are listed in the tables. MM, molecular mass.

Recently, we demonstrated selective complex formation of *Pseudomonas* *putida* cytochrome P450 (P450cam) with its redox partner proteins, putidaredoxin (PdX) and putidaredoxin reductase (PdR), using proliferating cell nuclear antigen (PCNA) subunits from *Sulfolobus solfataricus* as a self-assembling scaffold [13] (Figure 1A). *S. solfataricus* PCNA is a heterotrimeric ring-shaped protein that provides a scaffold for DNA-related enzymes by encircling dsDNA [14–16]. The PCNA heterotrimer is composed of three distinct subunits, PCNA1, PCNA2 and PCNA3, which can be separately expressed as monomer proteins and then assembled to form the heterotrimer. Thus, three genetic fusion proteins of the PCNA subunits with PdR, PdX and P450cam, i.e. PCNA1-PdR, PCNA2-PdX and PCNA3-P450cam, respectively, were used to form a heterotrimeric complex termed “PUPPET” (PCNA-utilized protein complex of P450 and its two electron transfer-related proteins). PUPPET showed much higher monooxygenase activity than an equimolar mixture of free PdR, PdX and P450cam. This is because PdR, PdX and P450cam are located in close proximity to each other on the PCNA heterotrimer and thus electrons are more efficiently relayed from PdR to P450cam via PdX. However, the peptide linkers between the PCNA subunits and the three enzymes, which are likely to affect the overall activity of PUPPET, were not examined in this earlier study.

In this report, we examined the effect of the peptide linker between PCNA2 and PdX on the monooxygenase activity of PUPPET. An optimum poly-L-proline-rich linker increased the activity 1.9-fold compared with the GGGGSLVPRGSGGGGS linker used in the previous report. The increased activity conferred by the poly-L-proline-rich linker could not be achieved using the optimized Gly_4_-Ser repeating linker. The poly-L-proline-rich linker provided a rigid spacer between PdX and PCNA and thus may have enabled a spatially-appropriate arrangement for more efficient interactions between PdX and P450cam. Therefore, linkers comprising a rigid peptide sequence are likely to be the best candidates for fusing enzymes with protein scaffolds for the construction of multienzyme complexes.

## Materials and Methods

### Plasmid construction

The plasmids expressing PCNA2 protein fused to PdX were constructed by inserting DNA sequences encoding peptide linkers between the C-terminus of PCNA2 and the N-terminus of the Cys73Ser/Cys85Ser mutant of PdX. First, two *Sap*I sites were inserted by PCR between the genes encoding PCNA2 and the Cys73Ser/Cys85Ser mutant of PdX in pHSG+PCNA2-PdX [17], using two primers, 5’-GGATCCGCTCTTCAGGAGGTGGTGGCTCTATGTCTAAAG-3’ (forward) and 5’-GAATTCGCTCTTCTACCGCCGTCCGCGC-3’ (reverse). The DNA fragment, containing two *Sap*I sites between PCNA2 and PdX, was amplified from the resulting plasmid by PCR using two primers, 5’-TATTTCCAGGGCCATATGATGAAAGCCAAAGTGATCG-3’ (forward) and 5’-ATGGCCCTGGAAATACAGG-3’ (reverse). A modified pET-15b(+) (Novagen, Darmstadt, Germany), termed pET15T, which lacked a *Sap*I site and had a TEV protease recognition site between a thrombin recognition site and an *Nde*I site, was linearized by PCR using two primers, 5’-GGATCCGGCTGCTAACAAAG-3’ (forward) and 5’-TTAGCAGCCGGATCCTTACCATTGCCTATCGGG-3’ (reverse). The amplified DNA fragment was fused to the linearized plasmid using the In-Fusion enzyme (Clontech, Mountain View, CA, USA), to obtain p2SSX. The oligonucleotide sequences encoding (G_4_S)_n_ (n = 1–3) were inserted between the sequences encoding PCNA2 and PdX into p2SSX by PCR. The oligonucleotide sequences encoding (G_4_S)_n_ (n = 4–6) and G_4_S(P_5_)_n_G_4_S (n = 1–5) were inserted by ligating hybridized oligonucleotides with sticky ends into *Sap*I-digested p2SSX. The oligonucleotides used to insert the linkers are summarized in Table S1.

A gene encoding the PCNA3-P450cam fusion protein was inserted between the *Nde*I and *Bam*HI sites of pET-15b(+). The resulting plasmid, pET15+oP3C, was mutated by PCR using two primers, 5’-AGCACCCGTCGTGAATTTAACGTTCGC-3’ (forward) and 5’-TTCACGACGGGTGCTGCCGATAATGTT-3’ (reverse). The resulting plasmid, pET15+P3 N_106R_C, expressed PCNA3_N106R_-P450cam fusion protein.

### Preparation of PUPPETs

PCNA1-PdR and PCNA2-PdX fusion proteins were expressed and purified as described in a previous report [13], except that BL21 Star (DE3) pLysS (Invitrogen, Carlsbad, CA, USA) was used. PCNA3-P450cam fusion protein, in which PCNA3 had a N106R mutation that improved the solubility of the fusion protein but did not affect PUPPET formation (unpublished data), was expressed and purified as described in the above report, except that T7 Express *I* ^*q*^ (New England BioLabs, Ipswich, MA, USA) was transformed with pET15+P3 N_106R_C.

An approximately equimolar ratio of PCNA1-PdR and PCNA2-PdX and a slightly excess amount of PCNA3-P450cam were mixed and incubated on ice for 1 h. The mixture was subjected to size-exclusion chromatography on a Superdex 200 10/300 GL column (1.0 × 30 cm; GE Healthcare, Little Chalfont, Buckinghamshire, UK) with 50 mM potassium phosphate buffer, pH7.4, containing 150 mM potassium chloride and 2 mM d-camphor (Wako Pure Chemical Industries, Osaka, Japan). The fractions with an Abs_455_/Abs_392_ ratio of 0.31–0.34 were collected. The collected fractions were combined and concentrated using an Amicon Ultra-15 Centrifugal Unit (30,000 NMWL; Millipore, Billerica, MA, USA). The d-camphor was then removed using a HiTrap desalting column pre-equilibrated with 50 mM potassium phosphate buffer, pH7.4, containing 150 mM potassium chloride.

### Enzyme assays

Cytochrome c reduction rates were determined from the absorption changes at 550 nm. The extinction coefficient difference (cytochrome c^red^ – cytochrome c^ox^) used to calculate the rates was 21.1 mM^-1^ cm^-1^. The assays were performed in 50 mM potassium phosphate buffer, pH7.4, containing 150 mM potassium chloride, 20 µM cytochrome c (Sigma-Aldrich, St. Louis, MS, USA), 50 µM NADH (Sigma-Aldrich) and 4.5 nM heterotrimeric complex. P450cam catalytic activities were determined from O_2_ consumption rates, which were directly measured using a Clark-type oxygen electrode, by subtracting the values obtained in the absence of d-camphor from those in the presence of d-camphor. The assays were performed in 50 mM potassium phosphate buffer, pH7.4, containing 150 mM potassium chloride, 500 µM d-camphor, 100 µM NADH and various concentrations of the heterotrimers. All assays were conducted at 25°C.

## Results

### Construction of PCNA2-PdX fusion proteins

**Figure 2:**
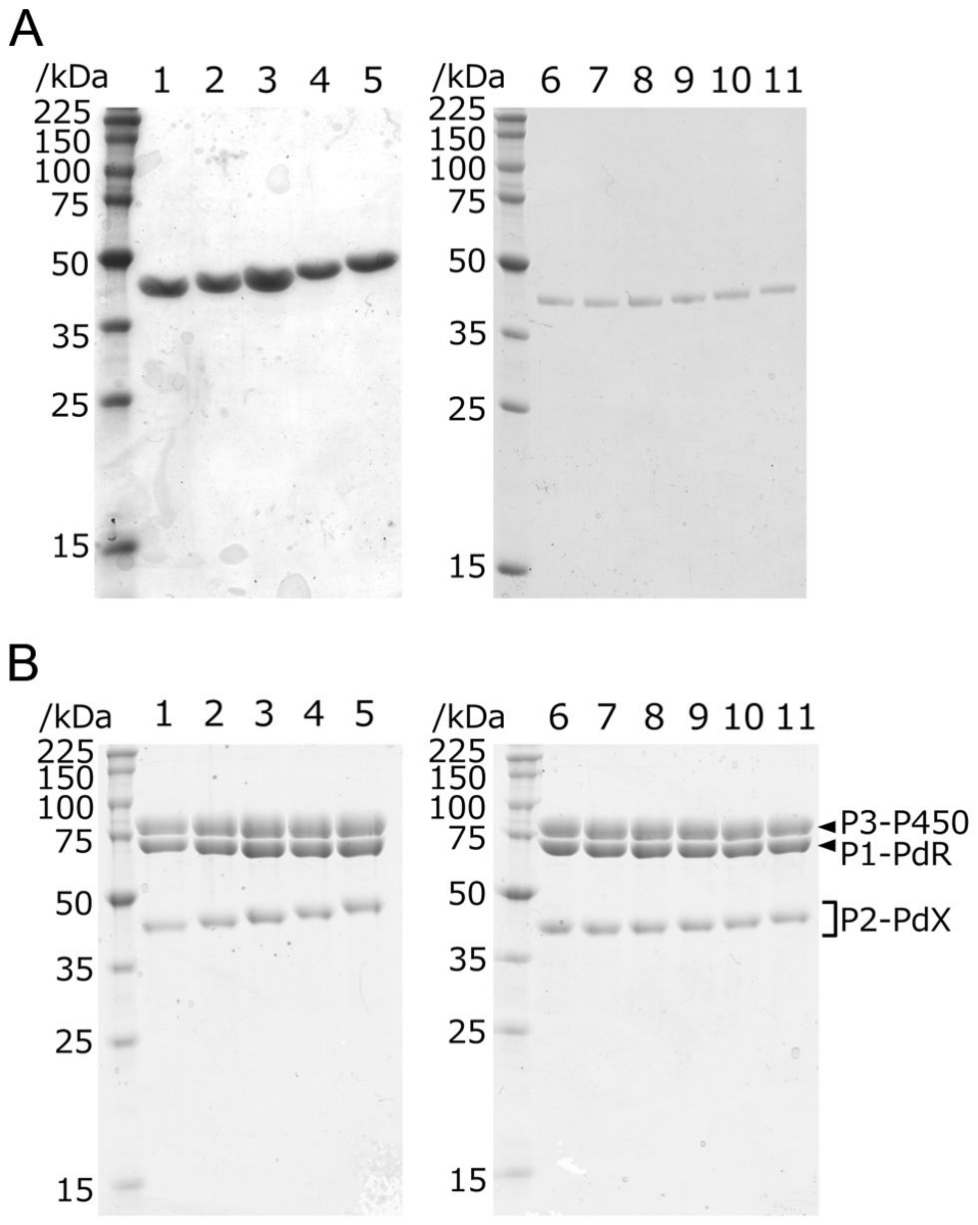
SDS-PAGE analyses of PCNA2-PdX fusion proteins and PUPPETs. (A) PCNA2-PdX fusion proteins: lane 1–5, PCNA2-G _4_S(P_5_)_n_G_4_S-PdX (n = 1–5); lane 6–11, PCNA2-(G_4_S)_n_-PdX (n = 1–6) (B) PUPPETs: lane 1–5, PUPPET-P_n_ (n = 1–5); lane 6–11, PUPPET-G_n_ (n = 1–6). P1-PdR, P2-PdX and P3-P450 are PCNA1-PdR, PCNA2-PdX and PCNA3-P450cam, respectively.

**Figure 3:**
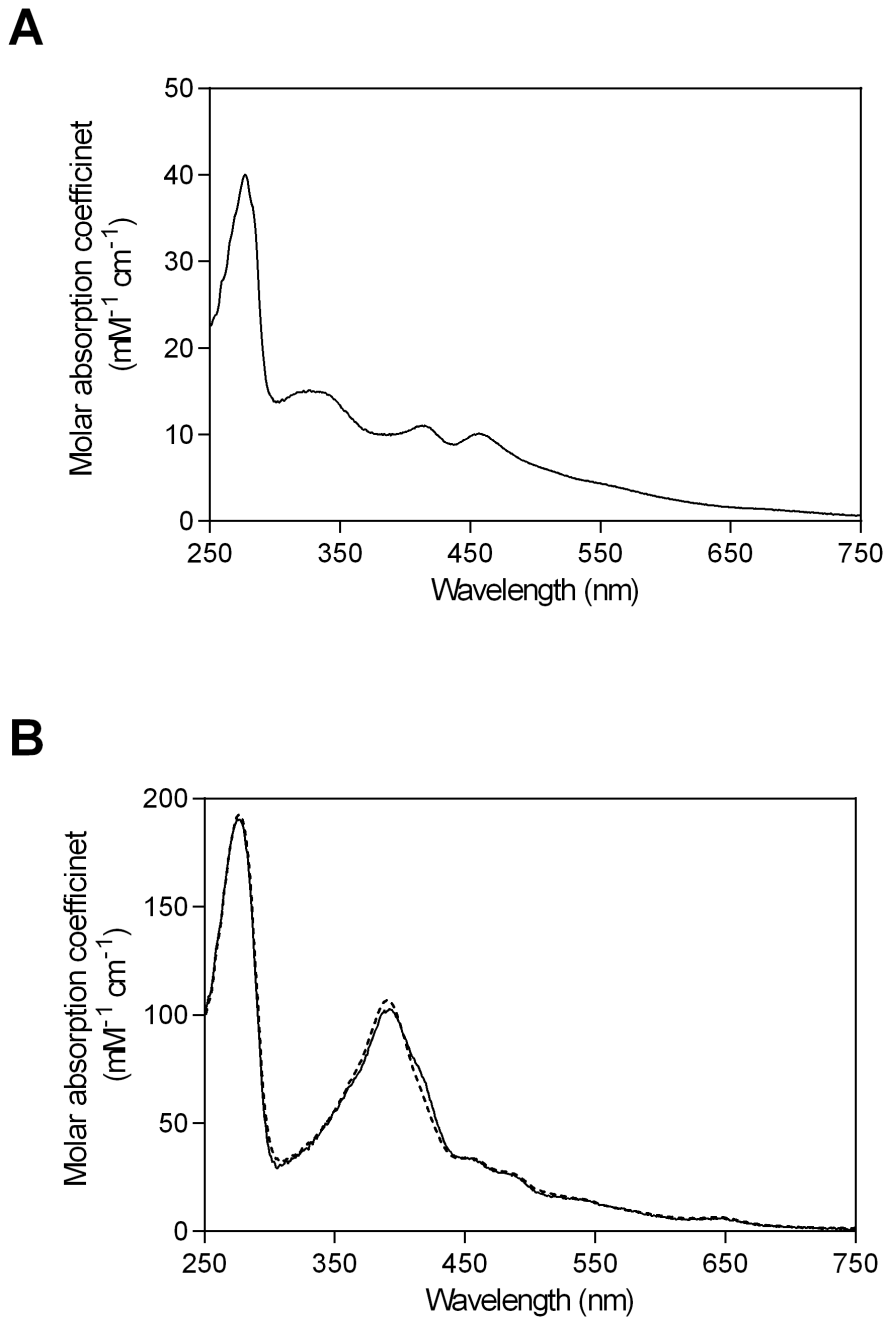
UV–Vis spectra of PCNA2-G _4_S-PdX and PUPPET-G_1_. (A) UV–Vis spectrum of PCNA2-G _4_S-PdX. The peaks at 314, 412, and 460 nm, and the broad band in the long wavelength region, are specific to [2Fe-2S] cluster-containing proteins. UV–Vis spectra of PCNA2-(G_4_S)_n_-PdX (n = 2–6) and PCNA2-G _4_S(P_5_)_n_G_4_S-PdX (n = 1–5) are shown in Figure S1. (B) UV–Vis spectra of PUPPET-G_1_ (solid line), and a linear combination of the individual component protein spectra (broken line). PUPPET-G_n_ (n = 2–6) and PUPPET-P_n_ (n = 1–5) are shown in Figure S2.

To enhance the activity of PUPPET, we optimized the peptide linker connecting PdX and PCNA2, because PdX interacts with both PdR and P450cam, and the spatial arrangement of PdX relative to these enzymes affects their interactions in the PUPPET complex. We therefore introduced various lengths of the poly-L-proline or Gly_4_-Ser repeating linkers between PCNA2 and PdX. Rigid poly-L-proline peptides have often been used as spacers [18–22]. Flexible Gly_4_-Ser repeating peptides have also frequently been used for antibody engineering [23] and bifunctional fusion protein construction [24–26]. PCNA2-PdX fusion proteins, PCNA2-G _4_S(P_5_)_n_G_4_S-PdX (n = 1–5) and PCNA2-(G_4_S)_n_-PdX (n = 1–6), which have proline-rich linkers, G_4_S(P_5_)_n_G_4_S, and Gly–Ser-rich linkers, (G_4_S)_n_, respectively (Figure 1B), were successfully expressed and purified (Figure 2A). The fusion proteins retained characteristic UV–Vis spectra for PdX (Figure 3A and Figure S1).

### Preparation of the heterotrimers

Heterotrimeric complexes of PdR, PdX and P450cam were prepared by mixing PCNA1-PdR, PCNA2-PdX and PCNA3-P450cam. Each resulting protein complex, PUPPET-P_n_ (n = 1–5) and PUPPET-G_n_ (n = 1–6), comprising PCNA2-G _4_S(P_5_)_n_G_4_S-PdX (n = 1–5) and PCNA2-(G_4_S)_n_-PdX (n = 1–6), respectively (Figure 2B), showed similar UV–Vis spectra to a linear combination of PCNA1-PdR, PCNA2-PdX and PCNA3-P450cam, as previously reported [13] (Figure 3B and Figure S2). The spectrum of each PUPPET had a slightly lower absorbance around 390 nm and slightly higher absorbance around 420 nm, compared with the linear combination spectra of PCNA1-PdR, PCNA2-PdX and PCNA3-P450cam as observed in the previous study. The linker between PCNA2 and PdX did not significantly affect the PUPPET spectrum (Figure 3B and Figure S2).

### Cytochrome c reduction activities of PUPPET

**Figure 4:**
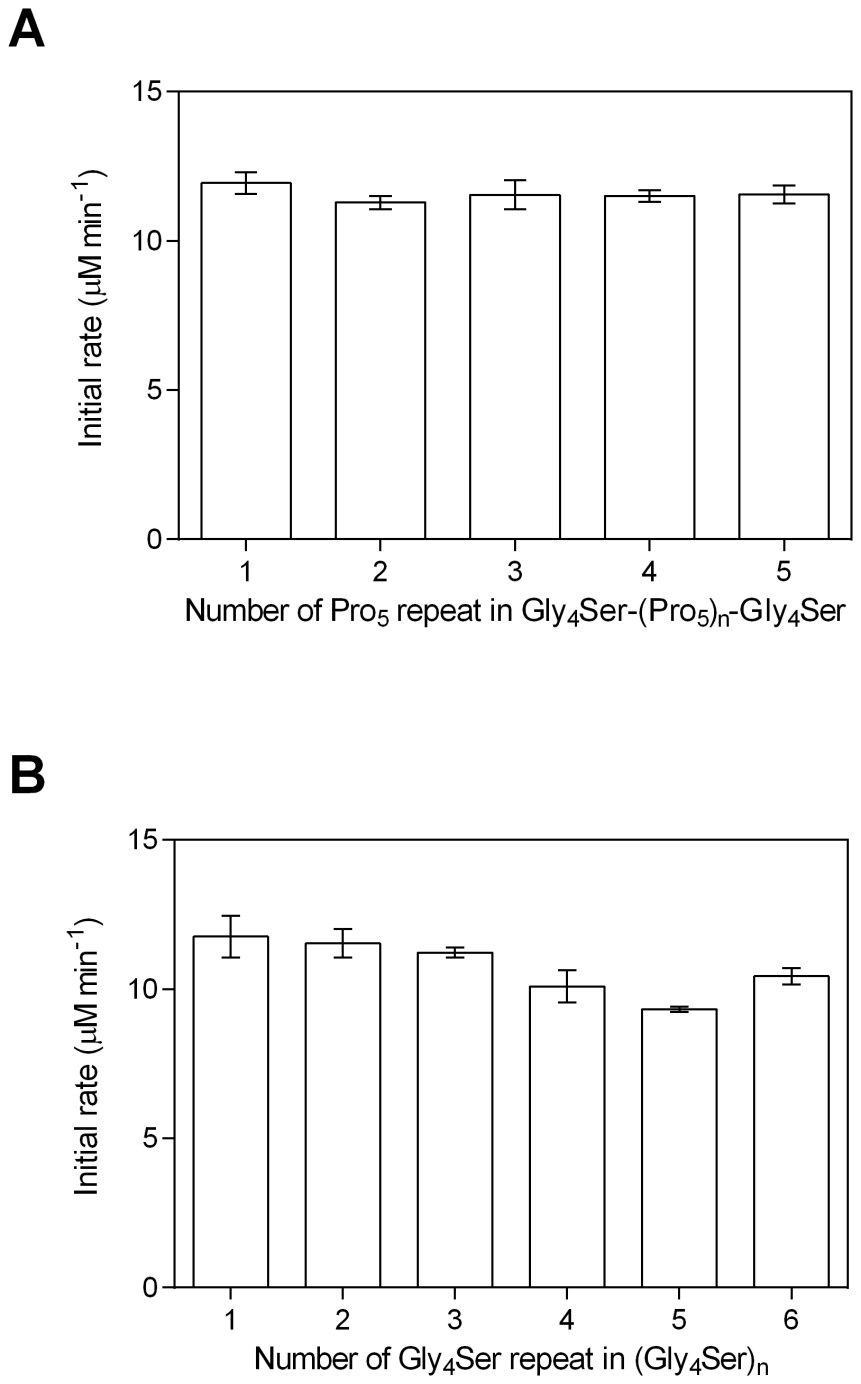
Cytochrome c reduction activities of the PUPPET linker variants. The activities of (A) PUPPET-P_n_ (n = 1–5) and (B) PUPPET-G_n_ (n = 1–6) were evaluated in a reaction mixture containing 4.5 nM enzymes and 20 µM cytochrome c. Error bars represent the standard errors of three replicates.

Cytochrome c reduction activities were measured to evaluate the effect of the linker on the electron transfer rate from PdR to PdX in PUPPET. The cytochrome c reduction rate reflects the electron transfer rate from PdR to PdX because direct cytochrome c reduction by PdR is negligible compared with that effected by reduced PdX [27]. The linker between PCNA2 and PdX was not observed to significantly affect the cytochrome c reduction activity of PUPPET (Figure 4).

### Effect of the linker on PUPPET activity

**Figure 5:**
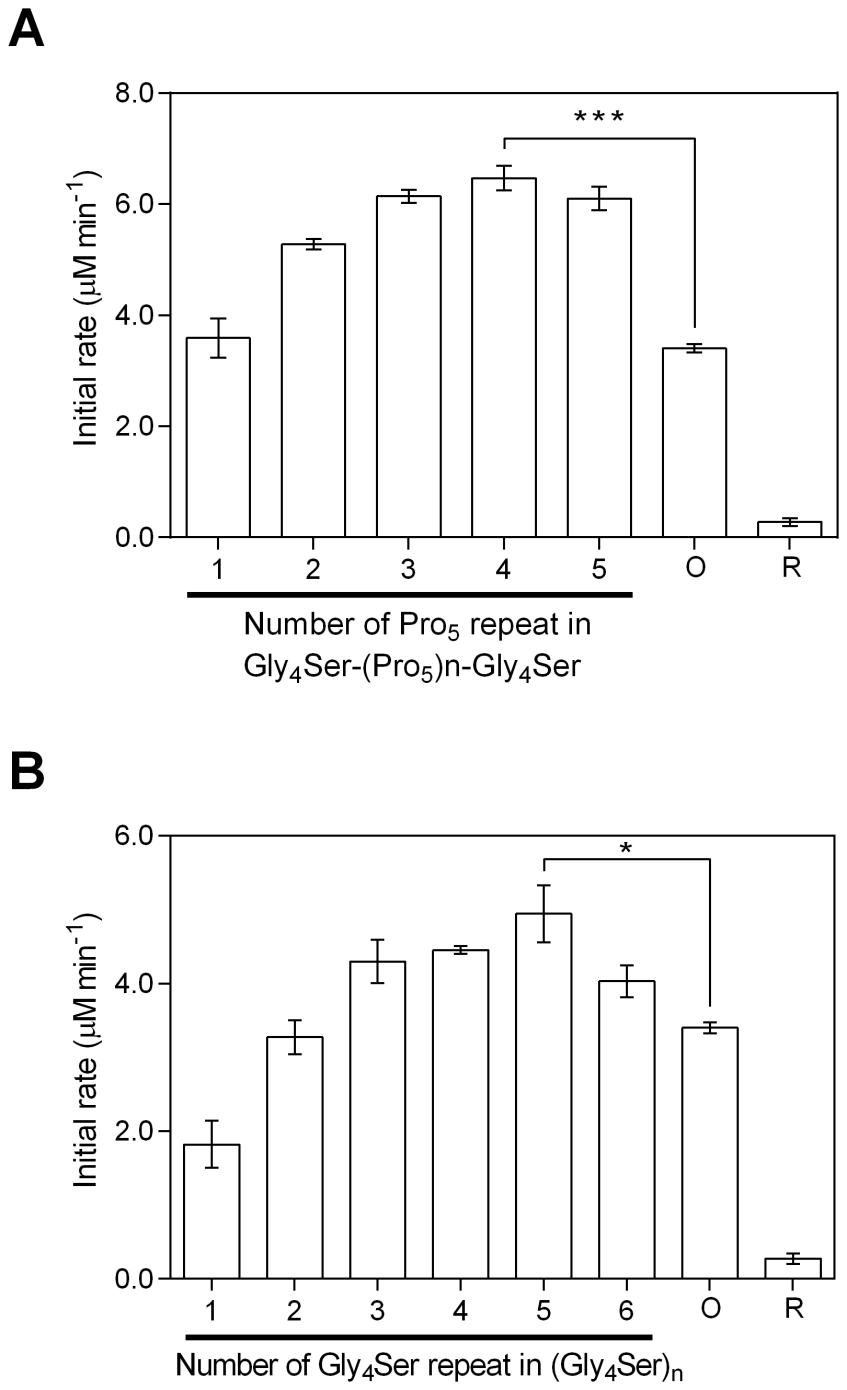
P450cam oxidation activities of the PUPPET linker variants. The activities of (A) PUPPET-P_n_ (n = 1–5) and (B) PUPPET-G_n_ (n = 1–6) were evaluated in reaction mixtures containing 18 nM enzyme. The activity of the previously reported PUPPET, containing a GGGGSLVPRGSGGGGS linker (indicated as “O”), and the activity of an equimolar mixture of PdR, PdX and P450cam (indicated as “R”), were also evaluated under the same reaction conditions. Error bars represent the standard error of three replicates; *P < 0.05, ***P < 0.001 (Student’s t-test).

The effect of the linker on the monooxygenase activities of PUPPET was evaluated by measuring the substrate-dependent O_2_ consumption rate at the complex concentration of 18 nM (Figure 5). In PUPPET-P_n_, the activity increased with increasing linker length in the range of n = 1–3, although PUPPET-P_n_ (n = 3–5) showed similar activity (Figure 5A). Likewise, in PUPPET-G_n_, the activity increased with increasing linker length in the range of n = 1–3, although PUPPET-G_n_ (n = 3–6) showed similar activity (Figure 5B). Although the linker length had an effect on the monooxygenase activity of both PUPPET-P_n_ and PUPPET-G_n_, the poly-L-proline-rich linkers, G_4_S(P_5_)_n_G_4_S (n = 2–5), were more effective than the Gly_4_-Ser repeating linkers in enhancing the activity of PUPPET. The G_4_SP_20_G_4_S linker gave the highest activity (6.5 ± 0.3 μM.min^-1^); a level not achieved by the Gly_4_-Ser repeating linkers, (G_4_S)_n_ (n = 1–6). This activity is 1.9-fold greater than that of the previously reported PUPPET complex that had a GGGGSLVPRGSGGGGS linker (3.4 ± 0.1 µM min^-1^).

**Figure 6:**
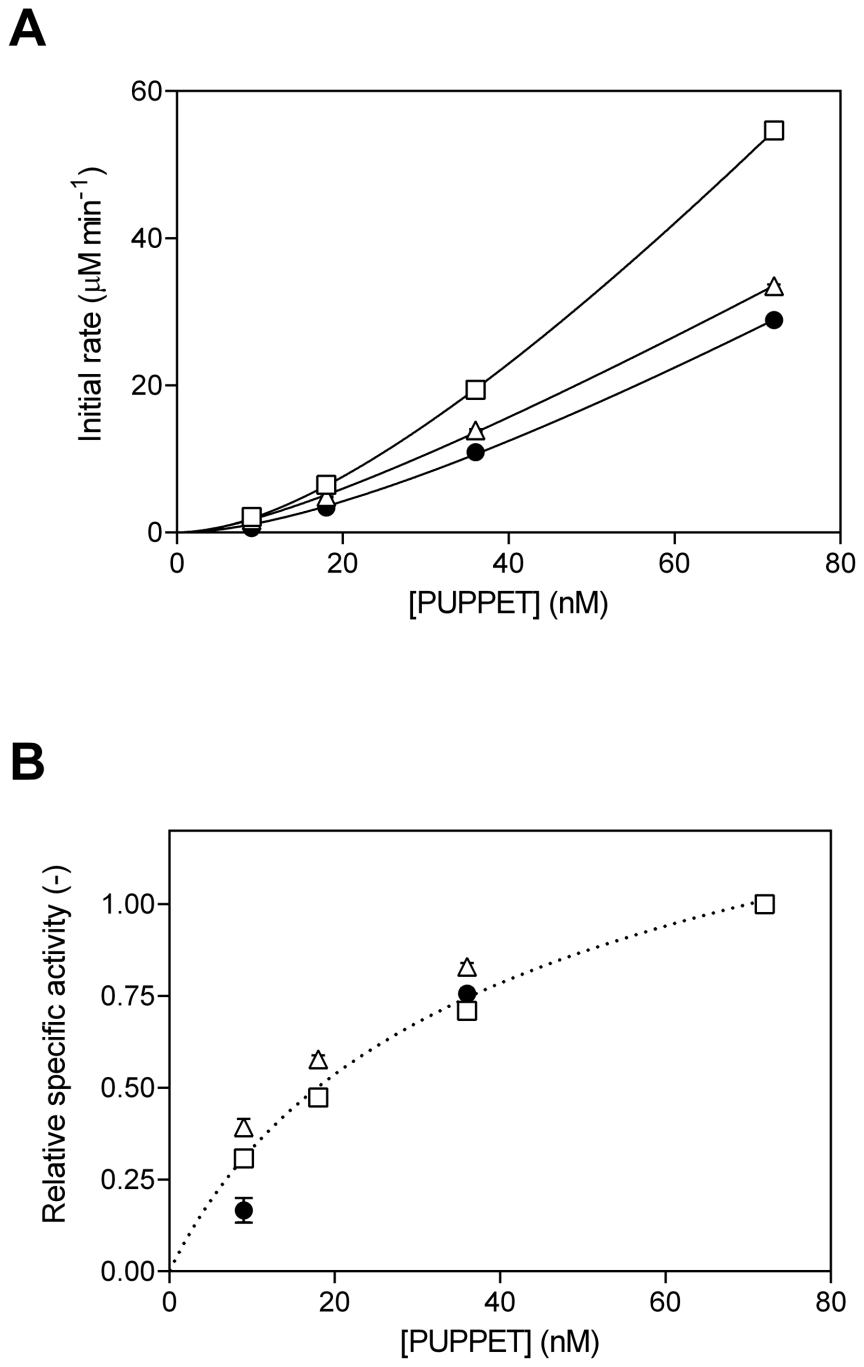
PUPPET concentration-dependent monooxygenase activities. (A) Initial rates and (B) specific activities were plotted against the protein concentration of PUPPET-P_4_ (open squares), PUPPET-G_5_ (open triangles) and original PUPPET (closed circles). Each specific activity was normalized using the activity at 72 nM in Figure 6B. Error bars represent the standard error of three replicates.

We compared the monooxygenase activities of PUPPET-P_4_ and PUPPET-G_5_ to the original PUPPET complex (Figure 6), using various enzyme concentrations. In agreement with the previous report [13], the initial activity of each PUPPET did not have a linear relationship with the PUPPET concentration (Figure 6A), and the apparent specific activity decreased according to the decrease in PUPPET concentration (Figure 6B), probably because of the dissociation of PCNA3-P450cam from PUPPET at lower PUPPET concentrations. At each enzyme concentration, PUPPET-P_4_ showed higher activity compared with PUPPET-G_5_. The apparent specific activities of PUPPET-P_4_ and PUPPET-G_5_ showed similar PUPPET concentration dependency to that of the original PUPPET, and therefore the enhanced activity levels of PUPPET-P4 and PUPPET-G5 compared with the original PUPPET complex are independent of the PUPPET concentration.

## Discussion

The functions of natural multi-domain proteins are often regulated by properties of the interdomain-connecting peptide linkers, such as their hydrophobicity and rigidity (reviewed in 28). The functions of artificial chimeric proteins are often similarly dependent on the linker properties [29]. Recently, artificial scaffold-mediated multienzyme complexes, in which linkers connect enzymes with single scaffolds, have attracted much attention because scaffolds can provide a basis for the precise control of the relative position and orientation of multiple enzymes [1]. Although the effect of linker length on protein-ligand and protein–protein associations [30,31] and bifunctional multienzyme complexes [26,32,33] has been well studied, only a few reports have investigated the effect of the linker length on artificial scaffold-mediated multienzyme complexes [8,9]. Moreover, the effect of the type of linker on the overall activities of artificial scaffold-mediated multienzyme complexes has never been examined. Linkers in artificial multienzyme complexes [3,10–12], which catalyze cascade reactions, might not have been considered to significantly affect the whole catalytic activities because cascade reactions do not depend on direct physical contact between enzymes. In this study, we examined the effect of the type and length of the peptide linker connecting PdX with the protein scaffold on the overall activity of the artificial protein complex comprising P450cam, PdX and PdR.

The monooxygenase activity of PUPPET was observed to depend on the characteristics of the linker between PCNA2 and PdX (Figure 5). This implies that the linker affected the electron transfer process from PdR to P450cam, because the electron transfer from PdX to P450cam during their transient complex formation is a rate limiting step in the P450cam catalytic cycle [34]. The electron transfer process from PdR to P450cam is divided into two steps: reduction of PdX by PdR, and electron donation from reduced PdX to P450cam. The cytochrome c reduction activity, which reflects the reduction rate of PdX by PdR, was not significantly affected by changing the PCNA2-PdX linker (Figure 4). The dependence of cytochrome c reduction activity on the linker was not identical to that of the monooxygenase activity. This result indicates that the linker mostly affected the electron donation step from reduced PdX to P450cam.

The linker length of the poly-L-proline-rich linker, G_4_S(P_5_)_n_G_4_S (n = 1–5), had a marked effect on the monooxygenase activity of PUPPET (Figure 5). Though the dissociation of PCNA3-P450cam from PUPPET is known to have a negative effect on the activity [13,17], the effect of the linker length was not involved in this dissociation, because the relationship between concentration and activity of PUPPET was not affected by the linker variants (Figure 6B). The poly-L-proline linker has been used as a “molecular ruler”, because poly-L-proline is expected to form an *all*-*trans* type II helix and the length of the helix can be predicted as approximately 3 Å per residue [21,35]. Recent studies demonstrated that the end-to-end distances of the poly-L-proline peptides are shorter than those of perfectly rigid type II proline helices, probably because of the small number of *cis* isomers interspersed in aqueous solutions of poly-L-proline peptides [36–39]. Nevertheless, the end-to-end distance was reported to increase with an increase in the number of proline residues, up to at least 24 amino acids [38]. Therefore, the repeat number of the Pro residue in the linker is expected to control the distance between PCNA2 and PdX, and to subsequently affect the spatial arrangement of PdX relative to P450cam in PUPPET-P_n_. The strong dependence of the monooxygenase activity on the repeat number of the Pro residue in the G_4_S(P_5_)_n_G_4_S linker indicates that the relative spatial arrangement of PdX governs the electron donation step from reduced PdX to P450cam in the PUPPETs.

**Figure 7:**
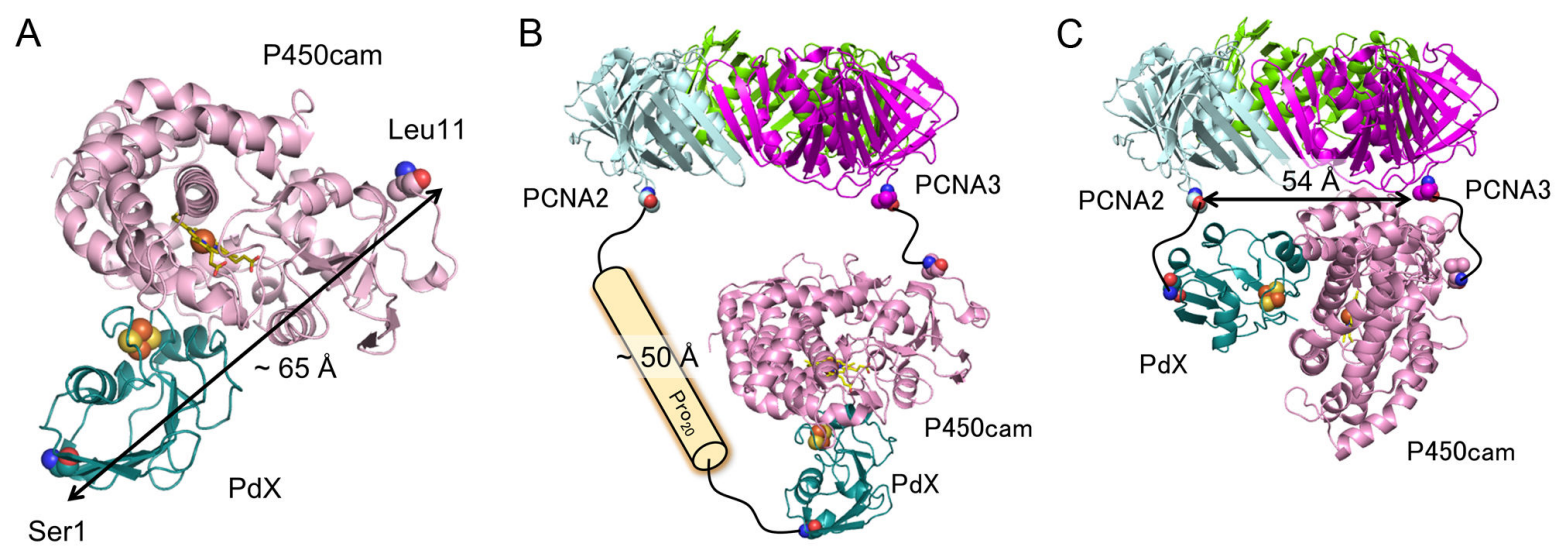
Models for binding between PdX and P450cam in PUPPET. (A) A docking model of P450cam and PdX. The docking program GRAMM-X [42] was used to generate the model from crystal structures of P450cam (PDB: 1DZ4) and PdX (PDB: 1XLP) according to a previous report [40]. (B, C) Spatial arrangement of P450cam and the PCNA ring when the PdX-binding site of P450cam faces (B) in the same direction as or (C) in a perpendicular direction to the PCNA ring. The distance between the C-termini of PCNA2 and PCNA3 was estimated from the crystal structure of the PCNA heterotrimer (PDB: 2NTI).

A previously reported docking model of the P450cam-PdX electron transfer complex [40], demonstrated that the distance between Leu11 of P450cam (of which residues Met1–Asn10 are undefined in the crystal structure), and the N-terminus of PdX, is approximately 65 Å in the electron transfer complex (Figure 7A). We also estimated that the mean end-to-end distance of helical linker Pro_20_ was approximately 50 Å according to a previous study [38]. PdX and P450cam are located on the same side of the PCNA ring because they are fused to the C-termini of PCNA2 and PCNA3, respectively. By combining the above structural information, we suggest a model of the electron transfer complex, in which the PdX-binding site of P450cam faces in a perpendicular direction towards the PCNA ring (Figure 7B). In this model, the G_4_SP_20_G_4_S linker between the C-terminus of PCNA2 and the N-terminus of PdX is the most effective in allowing PdX close to the P450cam binding site. On the contrary, the speculation that the binding site faces in a parallel direction to the PCNA ring as shown in Figure 7C, conflicts with the experimental data showing that the activity of PUPPET-P_n_ increased with an increase in the distance between the N-terminus of PdX and the C-terminus of PCNA2, in the range of n = 1–4. Therefore, the PdX-binding site of P450cam should preferably face in the perpendicular direction to the PCNA ring in the complex.

Although the length of the Gly_4_-Ser repeating linker, (G_4_S)_n_ (n = 1–6), also affected the monooxygenase activity, these linkers did not allow these PUPPET complexes to achieve the activity levels of even PUPPET-P_2_ (Figure 5). This result suggests that the mean end-to-end distances of the (G_4_S)_n_ linkers are shorter than that of G_4_SP_10_G_4_S. Evers et al. demonstrated that the elongation of a Gly- and Ser-rich (GGSGGS)_n_ linker from n = 1–6 only increased the distance between the two linked proteins by approximately 6 Å [41]. The Gly_4_-Ser repeating linker probably has similar characteristics to the (GGSGGS)_n_ linker. Therefore, the flexible (G_4_S)_n_ linkers do not enable PdX to be located at the appropriate distance away from the PCNA ring in order for the electron transfer complex to form, as shown in Figure 7B, and may instead cause PdX to interact with P450cam in an unfavorable manner, as shown in Figure 7C. The difference in the possible spatial arrangements of PdX in PUPPET-P_n_ and PUPPET-G_n_ might therefore result in the differences observed in their activities.

## Conclusion

In this study, we successfully enhanced the monooxygenase activity of the PCNA-mediated protein complex of PdR, PdX and P450cam by optimizing the peptide linker between PCNA2 and PdX. The linker affected the electron donation step from reduced PdX to P450cam in the complex. The G_4_SP_20_G_4_S linker most effectively enhanced the activity, most likely because the rigid Pro_20_ sequence contributed to PdX positioning close to the PdX-binding site of P450cam. On the other hand, the high activity obtained with this linker was not achieved with the flexible (G_4_S)_n_ linkers in the range of n = 1–6, owing to the short end-to-end distances of these flexible linkers. Recently, we have demonstrated that introduction of disulfide bonds between the PCNA subunits prevented the dissociation of PCNA3-P450cam from PUPPET and enhanced the activity [17]. Therefore, introduction of disulfide bonds can further enhaced the activity of PUPPET-P_4_. Our findings suggest that linkers containing rigid peptides are more suitable for the control of the spatial arrangement of enzymes in artificial multienzyme complexes in which component enzymes/proteins need to interact with each other, and can thereby result in improved catalytic activity.

## Supporting Information

Figure S1 **UV–Vis spectra of PCNA2-PdX linker variants.** UV–Vis spectra of PCNA2-G _4_S(P_5_)_n_G_4_S-PdX (n = 1–5, P_1_-P_5_) and PCNA2-(G_4_S)_n_-PdX (n = 2–6, G_2_-G_6_) are listed.
(TIF)

Figure S2 **UV–Vis spectra of PUPPET linker variants.** Solid lines indicate each PUPPET-P_n_ (n = 1–5, P_1_-P_5_) and PUPPET-G_n_ (n = 2–6, G_2_-G_6_) spectrum. Broken lines indicate a linear combination of the individual component protein spectra.
(TIF)

Table S1 **Oligo DNA sequences to insert peptide linkers between PCNA2 and PdX.**
(DOC)
