# Supplementary figures and images for: Fine Tuning of Spatial Arrangement of Enzymes in a PCNA-Mediated Multienzyme Complex Using a Rigid Poly-L-Proline Linker
Source: PLoS One. 2013 Sep 5;8(9):e75114. doi: 10.1371/journal.pone.0075114 (PMC3764174; doi:10.1371/journal.pone.0075114)

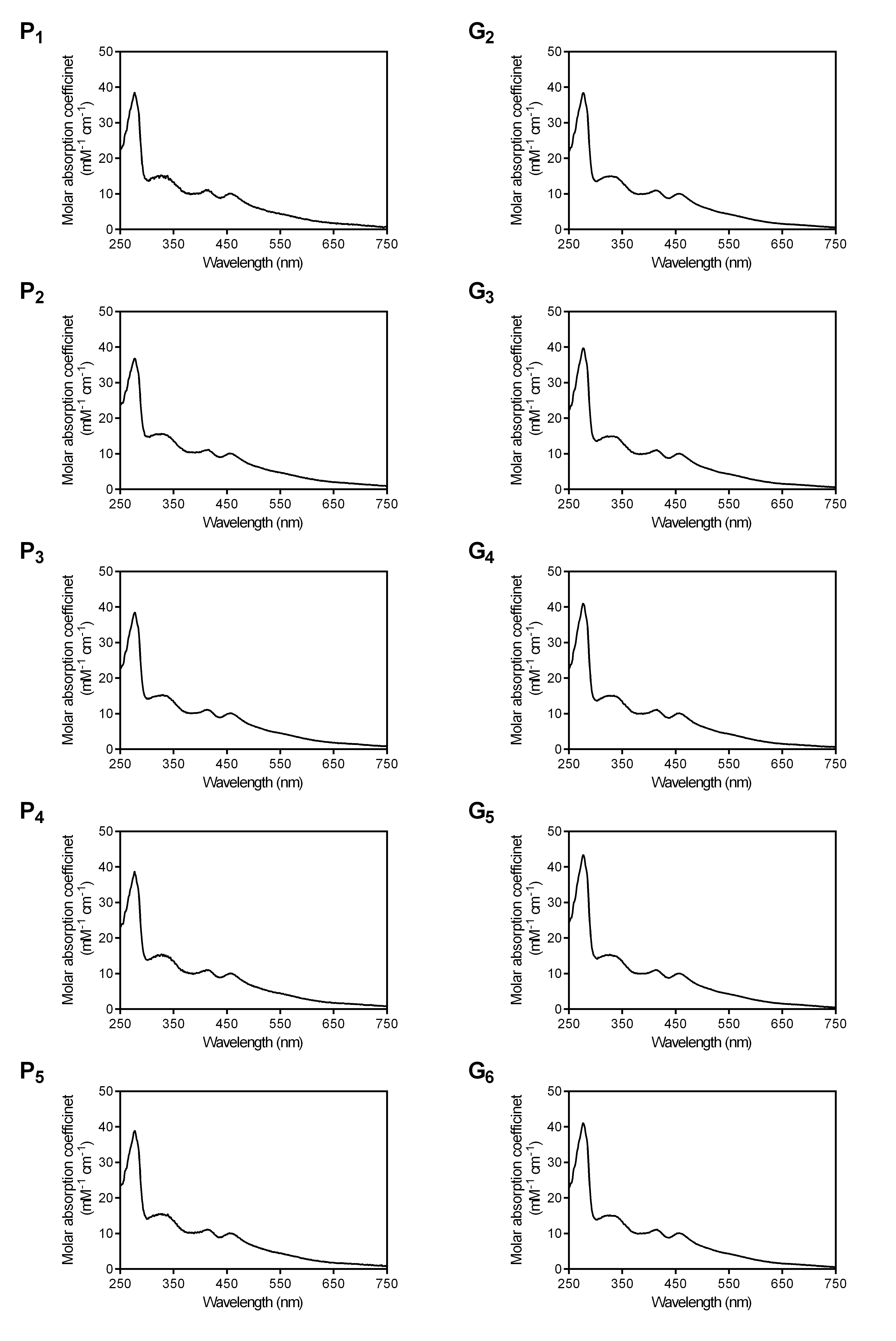

Supplement: Figure S1 — UV–Vis spectra of PCNA2-PdX linker variants. UV–Vis spectra of PCNA2-G 4S(P5)nG4S-PdX (n = 1–5, P1-P5) and PCNA2-(G4S)n-PdX (n = 2–6, G2-G6) are listed. (TIF) [file pone.0075114.s001.tif]

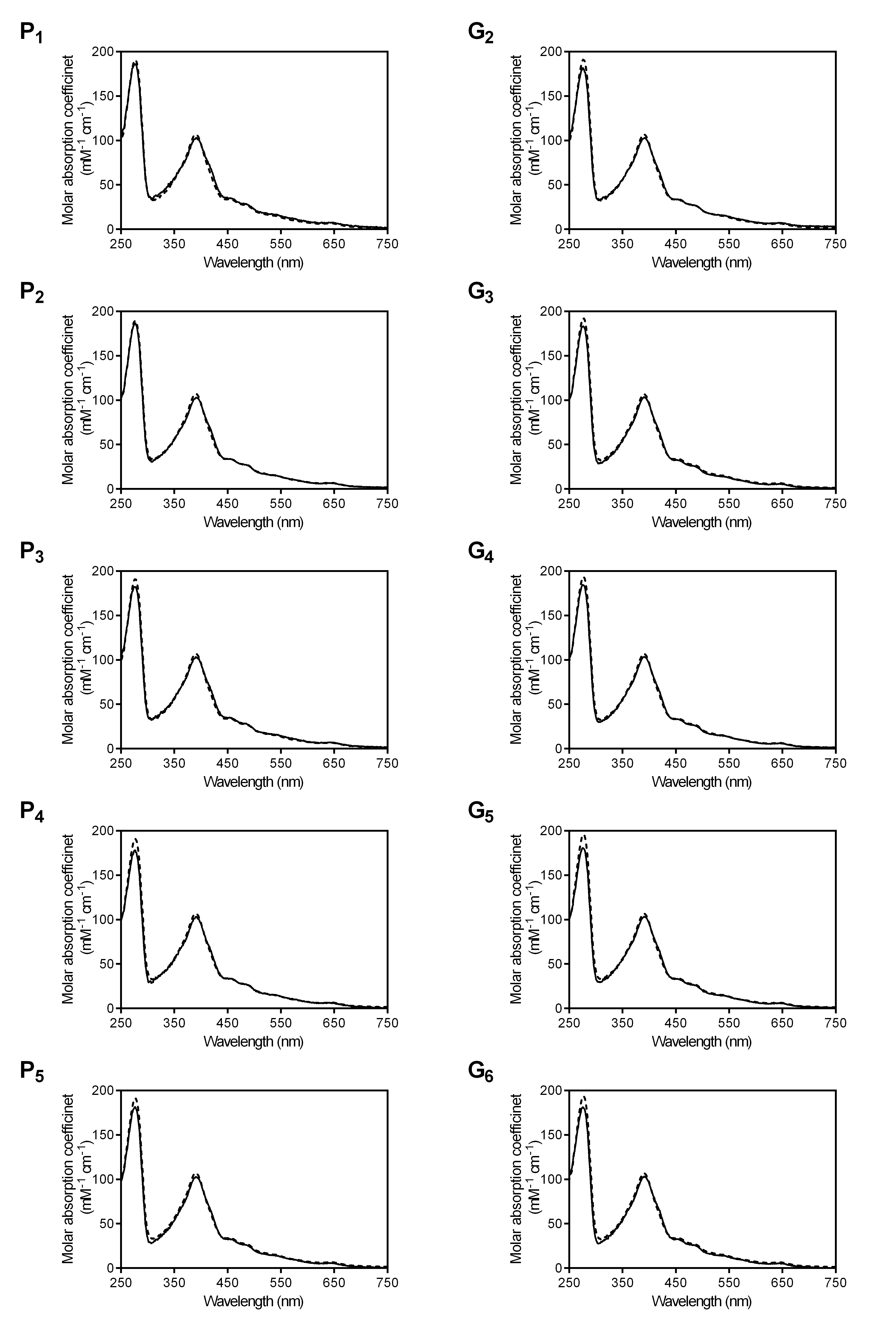

Supplement: Figure S2 — UV–Vis spectra of PUPPET linker variants. Solid lines indicate each PUPPET-Pn (n = 1–5, P1-P5) and PUPPET-Gn (n = 2–6, G2-G6) spectrum. Broken lines indicate a linear combination of the individual component protein spectra. (TIF) [file pone.0075114.s002.tif]
