# Supplementary material for: Fine Tuning of Spatial Arrangement of Enzymes in a PCNA-Mediated Multienzyme Complex Using a Rigid Poly-L-Proline Linker
Source: PLoS One. 2013 Sep 5;8(9):e75114. doi: 10.1371/journal.pone.0075114 (PMC3764174; doi:10.1371/journal.pone.0075114)
Supplement: Table S1 — Oligo DNA sequences to insert peptide linkers between PCNA2 and PdX. (DOC) [file pone.0075114.s003.doc]

**Table S1**. **Oligo DNA sequences to insert peptide linkers between PCNA2 and PdX.**

| Linker | Forward/reverse | Sequence |
| --- | --- | --- |
| (G_4_S)_1_ | Forward | 5’-GGAGGTGGTGGCTCTATGTCTAAAG-3’ |
|  | Reverse | 5’-GTCCGCGCGCGGTGC-3’ |
| (G_4_S)_2_ | Forward | 5’-GGAGGTGGTGGCTCTATG-3’ |
|  | Reverse | 5’-GCTTCCGCCACCGCCGTCCGCGC-3’ |
| (G_4_S)_3_ | Forward | 5’-GGTGGAGGAGGTTCTGGAGGTGGTGGCTCTATG-3’ |
|  | Reverse | 5’-GCTTCCGCCACCGCCGTCCGCGC-3’ |
| (G_4_S)_4_ | Forward | 5’-GGTGGCGGCAGCGGAGGAGGCGGTTCTGGCGGAGGTGGTAGC-3’ |
|  | Reverse | 5’-TCCGCTACCACCTCCGCCAGAACCGCCTCCTCCGCTGCCGCC-3’ |
| (G_4_S)_5_ | Forward | 5’-GGTGGCGGCAGCGGAGGAGGCGGTTCTGGTGGAGGAGGCTCTGGCGGAGGTGGTAGC-3’ |
|  | Reverse | 5’-TCCGCTACCACCTCCGCCAGAGCCTCCTCCACCAGAACCGCCTCCTCCGCTGCCGCC-3’ |
| (G_4_S)_6_ | Forward | 5’-GGTGGCGGCAGCGGAGGAGGCGGTTCTGGCGGTGGTGGAAGCGGTGGAGGAGGCTCTGGCGGAGGTGGTAGC-3’ |
|  | Reverse | 5’-TCCGCTACCACCTCCGCCAGAGCCTCCTCCACCGCTTCCACCACCGCCAGAACCGCCTCCTCCGCTGCCGCC-3’ |
| G_4_S(P_5_)_1_G_4_S | Forward | 5’-GGTGGCGGCAGCCCGCCACCTCCACCC-3’ |
|  | Reverse | 5’-TCCGGGTGGAGGTGGCGGGCTGCCGCC-3’ |
| G_4_S(P_5_)_2_G_4_S | Forward | 5’-GGTGGCGGCAGCCCGCCACCTCCACCGCCTCCTCCACCGCCC-3’ |
|  | Reverse | 5’-TCCGGGCGGTGGAGGAGGCGGTGGAGGTGGCGGGCTGCCGCC-3’ |
| G_4_S(P_5_)_3_G_4_S | Forward | 5’-GGTGGCGGCAGCCCTCCACCTCCACCGCCTCCTCCACCGCCACCACCGCCGCCTCCC-3’ |
|  | Reverse | 5’-TCCGGGAGGCGGCGGTGGTGGCGGTGGAGGAGGCGGTGGAGGTGGCGGGCTGCCGCC-3’ |
| G_4_S(P_5_)_4_G_4_S | Forward | 5’-GGTGGCGGCAGCCCTCCACCTCCACCGCCTCCTCCACCGCCACCACCGCCGCCTCCACCTCCACCGCCGCCC-3’ |
|  | Reverse | 5’-TCCGGGCGGCGGTGGAGGTGGAGGCGGCGGTGGTGGCGGTGGAGGAGGCGGTGGAGGTGGAGGGCTGCCGCC-3’ |
| G_4_S(P_5_)_5_G_4_S | Forward | 5’-GGTGGCGGCAGCCCTCCACCTCCACCGCCTCCTCCACCGCCACCACCGCCGCCTCCACCTCCACCGCCGCCACCTCCGCCTCCGCCC-3’ |
|  | Reverse | 5’-TCCGGGCGGAGGCGGAGGTGGCGGCGGTGGAGGTGGAGGCGGCGGTGGTGGCGGTGGAGGAGGCGGTGGAGGTGGAGGGCTGCCGCC-3’ |
